# Comparing Human-Smartphone Interactions and Actigraphy Measurements for Circadian Rhythm Stability and Adiposity: Algorithm Development and Validation Study

**DOI:** 10.2196/50149

**Published:** 2024-06-05

**Authors:** Hai-Hua Chuang, Chen Lin, Li-Ang Lee, Hsiang-Chih Chang, Guan-Jie She, Yu-Hsuan Lin

**Affiliations:** 1 College of Medicine Chang Gung University Taoyuan Taiwan; 2 Department of Family Medicine Chang Gung Memorial Hospital, Linkou Main Branch Taoyuan Taiwan; 3 Department of Industrial Engineering and Management National Taipei University of Technology Taipei Taiwan; 4 School of Medicine National Tsing Hua University Hsinchu Taiwan; 5 Department of Biomedical Sciences and Engineering, National Central University Taoyuan City Taiwan; 6 Department of Otorhinolaryngology - Head and Neck Surgery, Chang Gung Memorial Hospital, Linkou Main Branch Taoyuan Taiwan; 7 Institute of Population Health Sciences National Health Research Institutes Miaoli County Taiwan; 8 Department of Psychiatry National Taiwan University Hospital Taipei Taiwan; 9 Department of Psychiatry College of Medicine National Taiwan University Taipei Taiwan

**Keywords:** actigraphy, body composition, circadian rhythm, human-smartphone interaction, interdaily stability, obesity

## Abstract

**Background:**

This study aimed to investigate the relationships between adiposity and circadian rhythm and compare the measurement of circadian rhythm using both actigraphy and a smartphone app that tracks human-smartphone interactions.

**Objective:**

We hypothesized that the app-based measurement may provide more comprehensive information, including light-sensitive melatonin secretion and social rhythm, and have stronger correlations with adiposity indicators.

**Methods:**

We enrolled a total of 78 participants (mean age 41.5, SD 9.9 years; 46/78, 59% women) from both an obesity outpatient clinic and a workplace health promotion program. All participants (n=29 with obesity, n=16 overweight, and n=33 controls) were required to wear a wrist actigraphy device and install the Rhythm app for a minimum of 4 weeks, contributing to a total of 2182 person-days of data collection. The Rhythm app estimates sleep and circadian rhythm indicators by tracking human-smartphone interactions, which correspond to actigraphy. We examined the correlations between adiposity indices and sleep and circadian rhythm indicators, including sleep time, chronotype, and regularity of circadian rhythm, while controlling for physical activity level, age, and gender.

**Results:**

Sleep onset and wake time measurements did not differ significantly between the app and actigraphy; however, wake after sleep onset was longer (13.5, SD 19.5 minutes) with the app, resulting in a longer actigraphy-measured total sleep time (TST) of 20.2 (SD 66.7) minutes. The obesity group had a significantly longer TST with both methods. App-measured circadian rhythm indicators were significantly lower than their actigraphy-measured counterparts. The obesity group had significantly lower interdaily stability (IS) than the control group with both methods. The multivariable-adjusted model revealed a negative correlation between BMI and app-measured IS (*P*=.007). Body fat percentage (BF%) and visceral adipose tissue area (VAT) showed significant correlations with both app-measured IS and actigraphy-measured IS. The app-measured midpoint of sleep showed a positive correlation with both BF% and VAT. Actigraphy-measured TST exhibited a positive correlation with BMI, VAT, and BF%, while no significant correlation was found between app-measured TST and either BMI, VAT, or BF%.

**Conclusions:**

Our findings suggest that IS is strongly correlated with various adiposity indicators. Further exploration of the role of circadian rhythm, particularly measured through human-smartphone interactions, in obesity prevention could be warranted.

## Introduction

The global obesity epidemic has become a major public health concern in recent decades, and there is growing evidence to suggest that disrupted circadian rhythms and sleep disturbances are contributing factors [1,2]. Circadian rhythms are responsible for regulating energy homeostasis and adipose tissue metabolism, and disruptions to these rhythms have been associated with an increased risk of obesity [3]. Several animal studies [4], human studies with circadian gene variants [5,6], and examinations of shift workers have established this link [7]. However, most studies linking circadian disruptions and obesity have relied on self-reported questionnaires [8], which may be prone to bias and limitations. For instance, social jet lag, which is a well-known circadian rhythm indicator related to obesity, is quantified through self-reported measures of the difference in midsleep time between workdays and free days [8]. Accurate detection of circadian disturbances in clinical settings often necessitates continuous sleep-wake recordings or serial assays of timed plasma melatonin levels [9], which are more invasive and less commonly used.

Actigraphy, commonly used in research as a primary method for measuring physical activity and sleep patterns, operates on the principle that changes in body movements detected by an accelerometer are indicative of sleep onset and wakefulness. Traditionally, this approach has been seen as a “ground truth” for assessing circadian activity rhythms, especially when more invasive endogenous measurements like melatonin byproducts or urine secretion analysis are not feasible [10]. Additionally, recent studies have explored the effectiveness of consumer sleep-tracking devices in circadian measurement. Notably, devices such as the Fatigue Science Readiband (Fatigue Science), Fitbit Alta HR (Fitbit Inc ), EarlySense Live (EarlySense Ltd), ResMed S+ (ResMed Inc), and SleepScore Max (SleepScore Labs) have shown comparable or superior performance to actigraphy in certain sleep or wake measures [10], underscoring their emerging relevance in circadian rhythm research. While valuable as a proxy, capturing physical activity patterns that hint at circadian activity rhythms, actigraphy primarily reflects physical movement but not the entire spectrum of circadian biological rhythms. Moreover, the advent of modern technologies such as smartphones has challenged this traditional paradigm. Although actigraphy efficiently records physical motion, it falls short in capturing cognitive engagement that may occur with minimal physical movement, such as during smartphone use [11]. This limitation suggests a potential disconnect between physical movement and cognitive engagement, particularly in the context of contemporary technology use. Cognitive activities, especially those involving minimal physical movements like smartphone interactions, can significantly impact the sleep-wake cycle. These human-smartphone interactions not only reflect physical movements but also include social engagements and digital behaviors, thus offering a more comprehensive insight into an individual’s circadian rhythm in the modern era. They indicate social rhythms and present a broader spectrum of daily routines that contribute to our understanding of modern circadian rhythms. This approach challenges the traditional reliance on actigraphy alone for sleep tracking, which primarily measures physical activity. It underscores the need to reevaluate conventional methods and acknowledge the broader implications of cognitive and social dynamics on circadian activity rhythms.

In recent years, digital footprints, such as human-smartphone interactions, have emerged as a new way to observe human circadian rhythms [11,12]. Real-time, passively collected data from these interactions can provide long-term recordings of circadian rhythms in a natural setting, potentially offering an alternative to actigraphy. However, while some mobile apps can estimate sleep time based on human-smartphone interactions [11,12], no research has yet used these patterns to determine key circadian rhythm indicators like interdaily stability (IS) and intradaily variability (IV), which have been well established through actigraphy-based analysis [13]. Additionally, previous research [11,12] has only focused on healthy participants and has not been validated in patients with sleep disturbances or disrupted circadian rhythms, which are common comorbidities in obese individuals in clinical settings [14].

In this cross-sectional study, our primary goal was to investigate the relationship between adiposity and key indicators of sleep and circadian rhythm. We measured sleep-wake cycles using wrist-worn actigraphy, a well-established method in the field. In contrast, we also collected data on human-smartphone interaction patterns through our app, Rhythm, which automatically records smartphone use data and uses an algorithm similar to actigraphy to calculate the circadian rhythm of these interactions [13,15]. Our hypothesis was that the app-based measurements might offer insights differing from those provided by traditional actigraphy, potentially revealing unique associations with adiposity indicators.

The core objective of this study was to examine and compare the associations between various adiposity indices and sleep and circadian variables as measured by 2 different methods: traditional actigraphy and our novel app-based approach. Adiposity was indicated by BMI, visceral adipose tissue (VAT) area, and body fat percentage (BF%), while the sleep variables included total sleep time, sleep timing (chronotype), and the regularity of circadian rhythm. We aimed to assess how these relationships vary when circadian rhythms are measured through actigraphy as opposed to human-smartphone interactions. This comparative analysis was crucial for understanding the relative effectiveness of traditional and novel methods in reflecting the complex relationships between obesity, sleep, and circadian rhythms.

## Methods

### Study Population

A total of 78 participants (mean age 41.5, SD 9.9 years, 46/78, 59% women) were recruited from an obesity outpatient clinic and a workplace health promotion program at 7 hospitals in northern Taiwan for the study period between September 2021 and February 2023. The participants from the obesity outpatient clinics were assessed at baseline before commencing any weight reduction treatments. This is a crucial detail, as it implies that these patients have not yet started any therapeutic interventions that could potentially interfere with their adiposity or sleep, such as medications or other treatment modalities. This baseline assessment ensured that the data collected reflected their unaltered adiposity as well as their natural sleep and circadian rhythms without the influence of any treatments. Upon giving informed consent, all participants were asked to install the Rhythm app and wear a wrist actigraphy device for a minimum of 4 weeks, resulting in a total of 2182 person-days of data collection. Only participants with Android-operated smartphones were eligible to participate, with the condition that their phones were to be exclusively used by them during the study period. While participants were instructed to wear their actigraphy devices continuously, there was no specific guidance on the placement of their smartphones.

In determining the optimal sample size for this study, we focused on 2 key objectives. The primary objective was to explore the correlations between various adiposity indices, sleep, and circadian variables. The secondary objective involved comparing app-defined circadian rhythm and sleep indicators with actigraphy-derived measures across groups of individuals categorized as obese, overweight, and healthy controls. This required a detailed analysis at the level of person days.

We referred to a recent study [16] to guide our determination of sample size. That study used a similar methodology with 66 participants, including 33 patients with insomnia and 33 healthy controls, over a minimum duration of 4 weeks. This previous study [16] generated a comprehensive data set encompassing 2097 person-days and aimed to investigate the correlations between specific indicators and self-reported depressive symptoms, as well as sleep quality. It also sought to compare app-defined circadian rhythm and sleep indicators with actigraphy-derived measures.

This study consists of a total sample size of 78 participants, divided into 3 groups: obesity (n=29), overweight (n=16), and controls (n=33). The adequacy of our sample size is supported by sample size estimations for different outcome measures: When considering BMI as the main outcome and applying an *F* test with a fixed effect, omnibus, 1-way ANOVA model, with an α error probability of .05 and a statistical power of 0.90 for 3 groups, the total required sample size is 9. Similarly, if BF% is the main outcome, the same *F* test and ANOVA model, with the same α error probability and power, indicate a total required sample size of 15 for 3 groups. Additionally, when VAT serves as the main outcome, the sample size estimation under identical conditions indicates a total required sample size of 9 for 3 groups. Therefore, our current sample size for assessing adiposity-related outcomes is considered sufficient, as the effect sizes fall within the range of 0.44 to 2.59 and our statistical power exceeds 90%. This provides ample support for the suitability of this research study.

Surpassing the participant number [16] in the study we referenced, our larger sample size granted us the necessary statistical power to delve into the intricate relationships between multiple variables within our analytical framework. Additionally, the varied composition of our participant groups significantly strengthened the validity and applicability of our results. For the investigation of clinical correlations, we considered adiposity indices as dependent variables and identified several critical independent variables, including the (1) regularity of circadian rhythm, (2) physical activity level, (3) total sleep time, (4) sleep timing (chronotype), (5) age, and (6) gender. Adhering to the “1 in 10 rule,” which serves as a guideline for the number of predictor parameters in regression analysis [15], our sample size of 78 is adequately equipped to accommodate up to 6 independent variables as required by our analytical framework. This approach is validated by the principle that a sample size exceeding 60 is necessary for such analyses, confirming the adequacy of our chosen sample size.

### Sleep and Circadian Rhythm Measures: the Use of the Rhythm Mobile App and Actigraphy

#### Actigraphy Measurement

The participants were instructed to wear a research-grade wrist actigraphy device (MiCorTM A100, MiTAC Inc) on their nondominant wrist for a minimum of 4 weeks. The actigraphy device gathered acceleration data along 3 axes, sampled at 30 Hz, and calculated the Euclidean distance of the deviations from 0. The data was then bandpass filtered from 0.5-3 Hz, and 0 values above a predefined threshold were integrated within 2 seconds. From there, activity counts were derived by averaging the integrated segments over 1 minute [17,18]. For long-term recordings, we used a predefined threshold to identify “off-wrist” periods when the wristwatch might have been removed for activities like charging or showering. These “off-wrist” epochs were excluded from the analysis to mitigate potential impacts on rest-activity patterns caused by varying wearing habits. Any daily actigraphy data containing more than 6 hours of “off-wrist” time was discarded before further analysis.

The standard Cole-Kripke algorithm [17] was applied to the activity count data with slight modifications to determine the putative sleep and wake times. The algorithm categorizes data into rest and active states based on a weighted sum of the current minute and contiguous minutes, to minimize the impact of sudden changes in activity levels that could compromise the categorization. The algorithm was run on MATLAB software (MathWorks) and used preexisting codes.

#### Human-Smartphone Interaction Data Collection and Analysis

The app, Rhythm, was specifically designed to collect data on smartphone use by tracking 3 key variables: screen on or off events, notifications, and the app being used [12,19]. The data were collected in the background without interfering with the smartphone’s operation or significantly affecting battery life (less than 1%). To accurately capture app use behaviors in real-life scenarios and consider instances where users might temporarily leave their smartphones during activities like work or charging, we introduced the “app-count” method. This approach involves using longer durations to represent app use behaviors. Specifically, the “app-count” was defined as the sum of minute-by-minute use counts within nonoverlapping 5-minute epochs (288 epochs per day). By using these longer durations, we aimed to encompass the overall engagement with the smartphone, accounting for both periods of active use and temporary disengagement. Consequently, the number of apps used per minute was aggregated into these 5-minute epochs that did not overlap to eliminate excessive zero-count segments. These data were then used to mimic the activity data obtained from the wrist actigraphy device, estimate the near-24-hour cycle of the circadian rhythm, including the active and inactive phases, and determine sleep time during the inactive phase based on a threshold of app-counts.

#### Circadian Rhythm and Sleep Indicators

**Figure 1:**
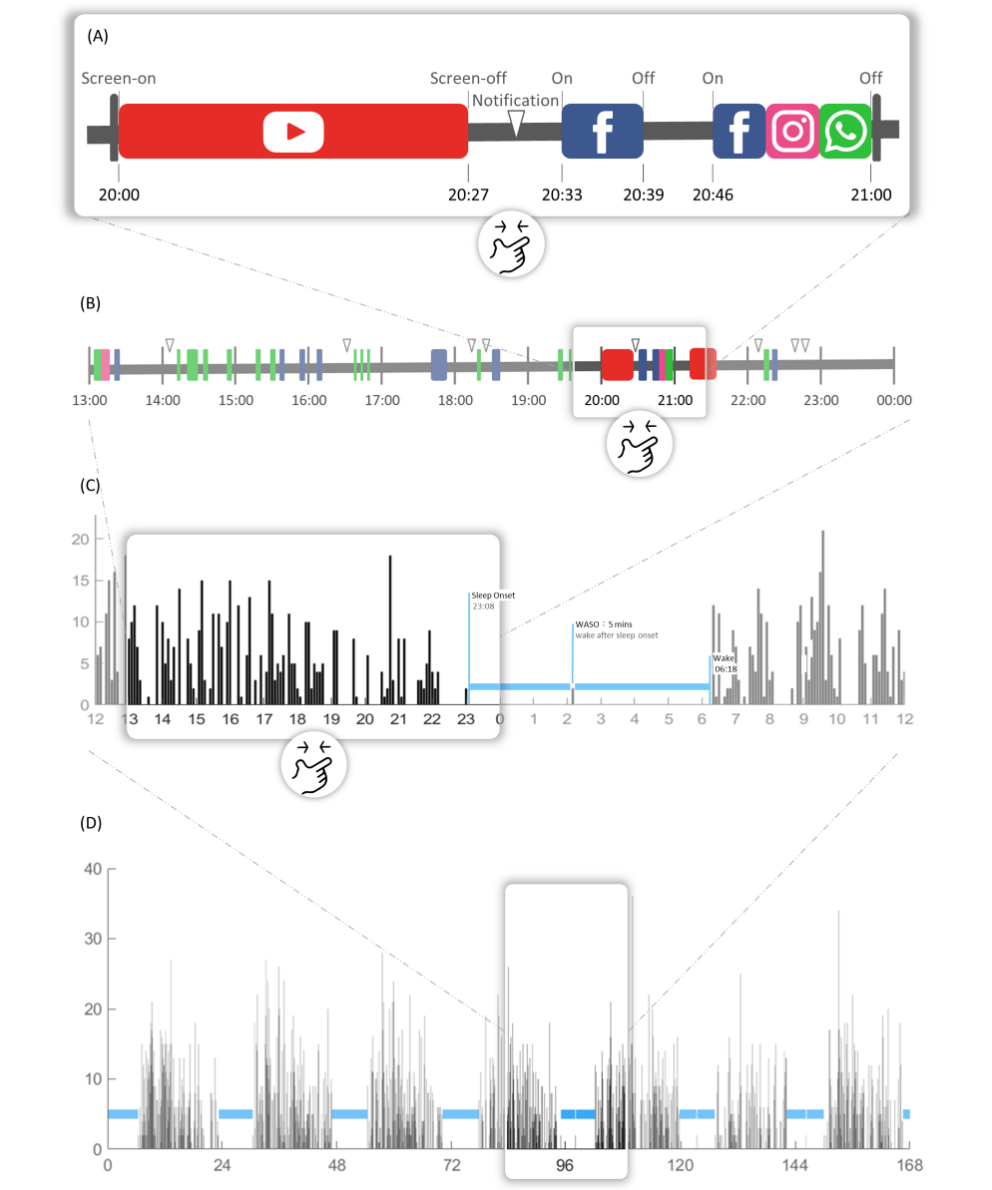
Algorithm for app-measured circadian rhythm and sleep indicators. (A) Timestamps of smartphone events recorded by the Rhythm app. (B) Deriving usage count from screen events. (C) Computing circadian rhythms and sleep patterns through smartphone. (D) Computing circadian rhythms with app-count analysis.

The acti-counts and app-counts generated from actigraphy, and smartphone use data were processed to determine 5 sleep indicators and 2 circadian rhythm indicators (Figure 1). The daily sleep indicators included sleep onset, wake time, midpoint of sleep, wake after sleep onset (WASO), and total sleep time (TST). The data were filtered to extract approximately 16 to 24 hour-long cycles, and putative sleep was assumed to occur at the half-cycle with nadir. Sleep onset was defined as a period of 8 consecutive epochs with a zero app-count, while wake time was defined as a period of 6 consecutive epochs with a nonzero app-count.

**Figure 2:**
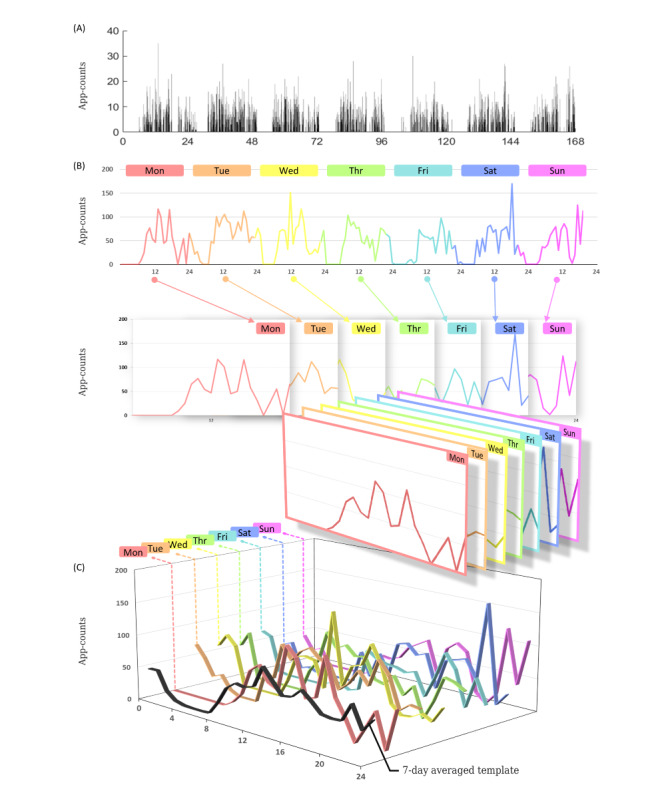
(A) The calculation of interdaily stability. (B) The hourly mean of the app count is calculated from the raw data and computed for each day of the recording period. (C) The mean of all hourly means across days is calculated, resulting in the grand mean.

The nonparametric method was used to calculate 2 circadian rhythm indicators, IS and IV (Figure 2). IS quantified the stability of the rhythms between days, that is, the coupling strength of the rhythms to the supposedly stable environmental factors. It could vary between 0 and 1, with higher values indicating more stable daily rhythms. IV indicated the fragmentation of the rhythms, that is, the frequency and extent of transitions between rest and activity. It could vary roughly between 0 and 2, with higher values indicating higher fragmentations [13].

The formula for IS is as follows:



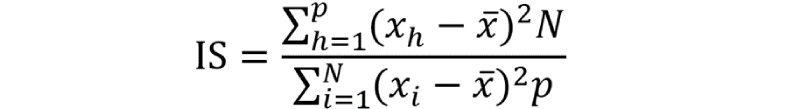



in which N is the total amount of data, p is the number of data point per day; x_h_ is the hourly means, x_i_ represents the individual data points, and 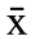 represents the mean of all days.

In this study, we chose the nonparametric method over cosinor analysis for several key reasons. First, cosinor analysis is typically applied to physical activity data under the assumption of relatively stable circadian rhythms. However, our research focused primarily on the association between disrupted circadian rhythms and adiposity. In this context, many of our participants likely exhibited disrupted circadian rhythms, under which even the data collected through actigraphy might not conform to the norms expected in cosinor analysis. Recent studies exploring circadian rhythm as a marker for various diseases have increasingly favored nonparametric or extended-cosine methods over traditional cosinor analysis in similar scenarios [20,21].

Furthermore, our app-based measurements transformed screen events from human-smartphone interactions into app-counts, similar to actigraphy’s acti-counts. However, these app-counts fundamentally differed in nature. Unlike acti-counts, which are derived from physical activity and characterized by continuous variations, app-counts did not inherently possess such continuity. Coupled with the issue of disrupted circadian rhythms in this study population, this complication made our data less suitable for cosinor analysis. The noncomparable units of most cosinor indicators and the unique, potentially nonstandard distribution of app-counts based on human-smartphone interactions reinforced our decision to opt for a nonparametric approach. Additionally, we did not use relative amplitude due to the unvaried nature of such measurements when derived from human-smartphone interactions.

### Physical Activity

Physical activity level was measured with an accelerometer to quantify the daily movements of each participant. Key features were calculated for each day, including the M10 and L5 values, representing the 10 hours of a day when the participant was most active and the 5 hours when they were least active, respectively. These are widely recognized indicators of a person’s circadian activity patterns [22,23]. To determine the M10, a 10-hour moving average was used to estimate the period of the day with the highest average acceleration, which was considered to be the participant’s overall physical activity level.

### Pittsburgh Sleep Quality Index

The Pittsburgh Sleep Quality Index (PSQI) was used to measure the overall sleep quality of participants in one month. This index comprises 19 items, evaluating 7 components of sleep quality, such as subjective sleep quality, sleep onset latency, total sleep duration, sleep efficiency, sleep disturbance, use of sleep medication, and daytime dysfunction. The sum of the 7 component scores yields one total score of subjective sleep quality (range 0-21); higher scores represent poorer subjective sleep quality. The cutoff score for PSQI-defined cases of poor sleep quality is a 6 or greater [24]. A Taiwanese version of the PSQI had been validated with adequate reliability [25].

### Anthropometric Measurements and Weight Status

All participants had their weight (in kilograms) and height (in centimeters) measured without shoes, following standard protocols. The BMI was calculated by dividing the body weight (in kilograms) by the square of the body height (in meters), resulting in units of kg/m^2^. The BMI categories used were overweight, defined as a BMI between 24 kg/m^2^ and less than 27kg/m^2^, and obesity, defined as a BMI equal to or greater than 27 kg/m^2^ [26].

### Body Composition Measurements

A total of 2 techniques were used to gather body composition measurements of the participants. For patients in the obesity outpatient clinic, dual-energy fan-beam X-ray absorptiometry (Hologic Horizon DXA system) was used with an array scan mode, following the manufacturer’s protocol for body composition measurements. The Hologic APEX software (version 5.6.0.4) was used to assess the scans. Each participant was placed according to the guidelines set by the International Society for Clinical Densitometry to ensure accuracy and consistency [27]. For individuals enrolled in the workplace health promotion program, body composition was measured using bio-electrical impedance analysis, which is based on the differences in the conductivity of various components of the human body. We used the IOI-353 BC analyzer (Jawon Medical) to evaluate segmental multifrequency impedance values at 1, 5, 50, 250, 550, and 1000 kHz with a tetra-polar 8-point tactile electrode system [28]. Measurements of BF%, total body fat, total lean mass, and VAT area were obtained.

### Statistical Analysis

The *χ*^2^ test and 1-way ANOVA were used to compare demographic variables and sleep and circadian rhythm indicators among the obesity, overweight, and control groups. Fisher least significant difference was used for further post hoc tests. A 2-way ANOVA was used to compare the app-measured circadian rhythm and sleep indicators with their actigraphy counterparts. It was also used to compare these indicators among participants with obesity, overweight, and healthy controls.

The relationship between obesity, sleep, and circadian rhythm indicators was analyzed using a multivariable regression model. This approach was chosen as the evening chronotype, shorter total sleep time, and unstable circadian rhythm were found to be interconnected due to potential shared underlying factors. BMI, VAT, and BF% were used as the dependent variables, while 3 circadian rhythm and sleep indicators were used as independent variables, including (1) TST, (2) midpoint of sleep, which served as a proxy for chronotype, and (3) IS, the circadian rhythm indicator. The choice of these indicators was influenced by previous research on obesity and circadian rhythm, which has primarily focused on the concept of social jet lag. IS was specifically selected as it is more closely related to social jet lag, which is calculated from the midpoint of sleep on weekdays and weekends. Other variables included in the multivariable regression model were age, gender, and physical activity level (M10).

*P*<.05 was considered to be statistically significant. Data arrangement and statistical analysis were performed using SPSS Statistics (version 25; IBM Corp).

### Ethical Considerations

The study was approved by the institutional review boards of Chang-Gung Memorial Hospital (202002452A3 and 202100434B0A3) and was conducted in accordance with the ethical principles outlined in the Declaration of Helsinki. Informed consent was obtained from each participant. Data were anonymized and stored with caution. Consent for publication will be obtained using our institutional consent form.

## Results

### Description of the Cohort

Table 1 presents the characteristics of the study participants. The 3 weight status groups differed significantly in BMI and body composition, including BF%, total body fat, total lean mass, and VAT area. The mean age of all participants was 41.5 (SD 9.9) years, and 59% (46/78) were women, with no significant differences in age or gender across the groups. The obesity group had significantly lower levels of physical activity on average (M10) than the overweight and healthy control groups. Both the obesity and overweight groups had average scores on the PSQI questionnaire above the cutoff point of 6, indicating poor sleep quality, whereas the healthy control group had an average PSQI score below the cutoff point.

**Table 1:** Demographic characteristics of participants in obesity, overweight, and control groups.

Characteristics		Obesity (n=29)	Overweight (n=16)	Control (n=33)	Chi-square (2)	*F* test (2)	*P* value	Effect Size	Comparison	
**Sex, n**										—^a^
	Male	10	7	15	0.830	—	.66	—		
	Female	19	9	18	—	—	—	—		
Age (years), mean (SD)		44.4 (9.9)	40.8 (10.4)	39.4 (9.3)	—	2.104	.13	—	—	
BMI (kg/m^2^), mean (SD)		30.8 (2.7)	25.3 (0.8)	21.5 (1.6)	—	169.951	<.001	2.59	Ob^b^>Ow^c^>C^d^	
Body fat %, mean (SD)		40.4 (6.4)	34.4(2.8)	27.6 (4.9)	—	46.850	<.001	1.16	Ob>Ow>C	
Total body fat (g), mean (SD)		32,237.2 (7012.5)	22,527.3 (2023.3)	15,532.2 (3496.2)	—	88.468	<.001	2.13	Ob>Ow>C	
Total lean mass (g), mean (SD)		45,804 (6636.6)	42,914.4 (4047.8)	40,324.1 (5555.2)	—	7.047	.002	0.44	Ob>C	
VAT^e^ area (cm^2^), mean (SD)		151.3 (40.2)	84.6 (18.5)	53.9 (23)	—	83.477	<.001	1.90	Ob>Ow>C	
Physical activity (M10^f^), mean (SD)		57.96 (26.58)	81.32 (17.71)	77.37 (22.55)	—	7.332	.001	0.45	Ob<Ow, C	
PSQI^g^ score		7.5 (4.2)	8.4 (4.6)	5.3 (2.7)	—	4.186	.02	0.31	Ow>C	

^a^Not applicable.

^b^Ob: obesity group.

^c^Ow: overweight group.

^d^C: control group.

^e^VAT: visceral adipose tissue.

^f^M10: the maximum 10 hours of physical activity within a 24-hour period (derived from actigraphy data).

^g^PSQI: Pittsburgh Sleep Quality Index.

### Differences Between App and Actigraphy

Table 2 presents a comparison of app-measured and actigraphy-measured circadian rhythm and sleep indicators. Results showed no significant difference between app-measured and actigraphy-measured sleep onset and wake time, as well as the midpoint of sleep. However, the app-measured WASO was found to be longer (13.5, SD 19.5 minutes) than the actigraphy-measured WASO, resulting in a longer actigraphy-measured TST of 20.2 (SD 66.7) minutes compared to the app-measured TST. The obesity group had a significantly longer TST than the overweight and control groups, regardless of the measurement method. App-measured circadian rhythm indicators, including IS and IV, were significantly lower than their actigraphy-measured counterparts. The obesity group also had significantly lower IS than the control group, whether measured by app or actigraphy.

In addition, the multivariable-adjusted model revealed a negative correlation between BMI and app-measured IS (*P*=.007), and a borderline significant negative correlation between BMI and actigraphy-measured IS (*P*=.06). BF% and VAT showed significant correlations with both app-measured IS and actigraphy-measured IS (Table 3). However, when IS was replaced with social jet lag in the model, there was no significant relationship between BMI, BF%, VAT, and either app-measured or actigraphy-measured social jet lag (Table 4). The app-measured midpoint of sleep showed a positive correlation with both BF% and VAT, whereas the actigraphy-measured midpoint did not demonstrate a significant correlation with either variable. Actigraphy-measured TST exhibited a positive correlation with BMI, VAT, and BF%, while no significant correlation was found between app-measured TST and either BMI, VAT, or BF%.

**Table 2:** Comparison of app-measured and actigraphy-measured circadian rhythm and sleep indicators in participants across obesity, overweight, and control groups.

Indicators				Obesity, mean (SD)		Overweight, mean (SD)		Control, mean (SD)	*P* value differences							Comparison
									Group^a^		Measurement^b^		Interaction^c^			
**Sleep indicators**																
	**Sleep onset^d^**								.002		.06		.09		Ob^e^, C^f^<Ow^g^	
		App	23.74 (1.01)		24.35 (1.14)		23.66 (0.83)									
		Actigraphy	23.11 (0.73)		24 (1.08)		23.75 (0.85)									
	**Wake time^d^**								.04		.50		.68		—^h^	
		App	7.37 (1.27)		7.23 (1.05)		6.91 (0.8)									
		Actigraphy	7.08 (0.99)		7.34 (1.18)		6.74 (0.89)									
	**Midpoint of sleep^d^**								.04		.17		.39		—	
		App	3.56 (1.09)		3.79 (1.05)		3.28 (0.69)									
		Actigraphy	3.1 (0.7)		3.67 (1.05)		3.24 (0.82)									
	**Wake after sleep onset (minutes)**								.76		<.001		.78		—	
		App	17.5 (27)		18.4 (16.4)		14.7 (14.7)									
		Actigraphy	2.4 (2.2)		4 (4.7)		3.1 (3.1)									
	**Total sleep time (minutes)**								<.001		.003		.03		Ob>Ow, C	
		App	440 (52.3)		394.3 (35.2)		420.5 (48.3)									
		Actigraphy	475.8 (63)		436.5 (51.1)		416.4 (36.4)									
**Circadian rhythm indicators**																
	**Interdaily stability**								.001		<.001		.95		Ob<C	
		App	0.17 (0.11)		0.22 (0.11)		0.25 (0.09)									
		Actigraphy	0.37 (0.12)		0.42 (0.12)		0.44 (0.11)									
	**Intradaily variability**								.54		<.001		.23		—	
		App	1.3 (0.3)		1.28 (0.26)		1.22 (0.23)									
		Actigraphy	0.91 (0.18)		0.81 (0.22)		0.92 (0.25)									

^a^The effect of health controls versus overweight versus obesity.

^b^The effect of app versus actigraphy.

^c^The interaction of group and measurement.

^d^Time are in day decimal time, for example, 23.50=23:30 PM.

^e^Ob: obesity group.

^f^C: control group.

^g^Ow: overweight group.

^h^Not applicable.

**Table 3:** Results of a multivariate linear regression analysis for IS^a^ on weight status and body composition indicators.

					IS		Midpoint^b^			TST^c^		M10^d^		Age		Gender		Adjusted *R*^2^	
**BMI**																			
	**App**																		0.213
		*β* (SE)	–0.342 (0.123)			0.188 (0.109)			–0.119 (0.119)		–0.282 (0.108)		0.196 (0.108)		0.029 (0.111)				
		*P* value	.007			.09			.33		.01		.07		.79				
	**Actigraphy**																		0.262
		*β* (SE)		–0.225 (0.118)				0.012 (0.107)	0.458 (0.124)		0.081 (0.135)		0.285 (0.107)		0.004 (0.096)				
		*P* value	.06			.91			<.001		.56		.009		.97				
**Body fat %**																			
	**App**																		0.408
		*β* (SE)	–0.293 (0.106)			0.278 (0.094)			0.024 (0.096)		–0.252 (0.095)		0.296 (0.093)		–0.158 (0.095)				
		*P* value	.008			.004			.82		.009		.002		>.99				
	**Actigraphy**																		0.410
		*β* (SE)	–0.292 (0.105)			0.011 (0.099)			0.417 (0.112)		0.101 (0.121)		0.367 (0.121)		–0.206 (0.093)				
		*P* value	.02			.91			<.001		.41		<.001		.03				
**Estimate visceral adipose tissue area**																			
	**App**																		0.421
		*β* (SE)	–0.305 (0.105)			0.188 (0.093)			–0.128 (0.104)		–0.364 (0.093)		0.458 (0.091)		0.087 (0.093)				
		*P* value	.005			.05			.22		<.001		<.001		.36				
	**Actigraphy**																		0.437
		*β* (SE)	–0.268 (0.103)			0.015 (0.098)			0.335 (0.106)		–0.039 (0.117)		0.536 (0.093)		0.062 (0.091)				
		*P* value	.01			.88			.002		.74		<.001		.49				

^a^IS: interdaily stability.

^b^Midpoint of sleep.

^c^TST: total sleep time.

^d^M10: the maximum 10 hours of physical activity within a 24-hour period (derived from actigraphy data).

**Table 4:** Results of a multivariate linear regression analysis for social jet lag on weight status and body composition indicators.

					Social jet lag		Midpoint^a^			TST^b^		M10^c^		Age		Gender		Adjusted *R*^2^	
**BMI**																			
	**App**																		0.139
		*β* (SE)	–0.111 (0.102)			0.238 (0.113)			0.031 (0.103)		–0.272 (0.114)		0.238 (0.109)		–0.026 (0.111)				
		*P* value	.32			.04			.78		.02		.04		.82				
	**Actigraphy**																		0.247
		*β* (SE)		–0.152 (0.099)				0.048 (0.109)	0.452 (0.122)		–0.040 (0.131)		0.264 (0.107)		0.023 (0.106)				
		*P* value	.14			.66			<.001		.75		.02		.83				
**Body fat %**																			
	**App**																		0.361
		*β* (SE)	–0.127 (0.095)			0.314 (0.097)			0.153 (0.100)		–0.238 (0.098)		0.332 (0.096)		–0.206 (0.097)				
		*P* value	.19			.002			.12		.02		.001		.04				
	Actigraphy																		0.379
		*β* (SE)	–0.181 (0.093)			0.057 (0.099)			0.411 (0.112)		–0.055 (0.110)		0.338 (0.097)		–0.185 (0.097)				
		*P* value	.05			.57			<.001		.63		.001		.06				
**Estimate visceral adipose tissue area**																			
	**App**																		0.370
		*β* (SE)	–0.135 (0.094)			0.225 (0.097)			0.006 (0.102)		–0.349 (0.099)		0.497 (0.095)		0.037 (0.097)				
		*P* value	.16			.02			.95		.001		<.001		.70				
	**Actigraphy**																		0.405
		*β* (SE)	–0.147 (0.090)			0.057 (0.096)			0.331 (0.109)		–0.182 (0.110)		0.508 (0.095)		0.078 (0.094)				
		*P* value	.11			.56			.003		.10		<.001		.41				

^a^Midpoint of sleep.

^b^TST: total sleep time.

^c^M10: the maximum 10 hours of physical activity within a 24-hour period (derived from actigraphy data).

## Discussion

This study found that among all the circadian rhythm and sleep indicators, IS, measured by either actigraphy or human-smartphone interaction, presented with the most correlations with adiposity indicators, such as BMI, VAT, and BF%. These correlations remained significant even after adjusting for confounding factors, including TST, chronotype, physical activity level, age, and gender. These findings were consistent with previous studies that have examined the relationship between circadian rhythm, chronotype, and adiposity simultaneously, with circadian rhythm being the most significant indicator [29-31]. Some studies have measured the correlation between adiposity and circadian rhythm using IS measured through actigraphy [29,32,33], while others have relied on self-reported measures of social jet lag [8].

IS is a concept similar to social jet lag [33], but it provides a more comprehensive understanding of a person’s sleep patterns compared to social jet lag (Figure 2). Unlike social jet lag, which simply calculates the difference between sleep midpoint on workdays and days off [8], IS considers the regularity of sleep and wake times within a 24-hour period for a minimum of 7 days, including both workdays and days off. IS uses the variance method to quantify the consistency of a person’s sleep and wake times, while social jet lag is determined through questionnaires that reflect its limited scope. Accurately computing IS requires continuous 24-hour monitoring for at least 7 days [13], which can be done through methods such as actigraphy or mobile app use, as seen in this study. However, social jet lag can be evaluated through a questionnaire because it only requires limited information.

Although the data used to determine IS can also be used to calculate social jet lag, the reverse is not necessarily true. This study revealed that IS recorded through a mobile app had several significant correlations with the obesity index, whereas social jet lag did not, and its results were similar to those obtained through actigraphy. While the small sample size in the study may not reflect the correlation between social jet lag and obesity found in previous large-scale epidemiological studies, it highlights that when it comes to personalized and precise weight management, IS is a more precise and sensitive indicator than social jet lag.

In this study, we compared app-based measurements of circadian rhythm and sleep indicators with those obtained through actigraphy. Our findings align with previous research, showing typical results for app-based measurements [29,32-39]. However, actigraphy measurements diverged, either failing to demonstrate similar results (Table 3) or presenting inconsistent findings with established patterns in previous studies. This highlights the potential superiority of the app in capturing circadian rhythms more accurately, especially considering its consistency with established research and ability to track broader circadian dynamics.

Notably, this study revealed several key differences between the 2 methods. First, the app’s internal sleep measurements showed a significant correlation with BMI, while actigraphy’s IS correlation with BMI was only marginally significant (*P*=.06). Second, the app identified an association between the evening chronotype (measured by sleep midpoint) and both BF% and VAT, a connection not found with actigraphy. Third, TST measured through actigraphy positively correlated with BMI, VAT, and BF%, contrary to app measurements and previous studies suggesting that sufficient sleep time is generally linked with lower obesity rates [40-42]. Interestingly, this study’s average sleep time was 7 hours, fitting within the substantial evidence that sleeping less than 7 hours a night can lead to adverse health consequences, including obesity [43].

Addressing concerns that the app might overestimate sleep time compared to actigraphy, our results showed no significant differences in sleep onset, wake time, or sleep midpoint between the 2 methods. The primary variance was in the WASO duration, leading to a longer TST as measured by actigraphy. However, this discrepancy had a minimal impact on circadian rhythm evaluation, as we adjusted for TST in our regression models to mitigate its influence on the association between circadian rhythm indicators and adiposity markers.

Intriguingly, while the actigraphy-measured TST was positively correlated with BMI, VAT, and BF%, the app-measured TST was not, indicating a complex relationship between sleep parameters and adiposity. Additionally, extensive research, including meta-analyses [44-46], suggests a complicated link between longer sleep duration and higher obesity risk in adults, influenced by age and other factors. Given that most sleep duration data in research is self-reported [44-46], adding another layer of complexity, our findings emphasize the importance of considering these nuances and the variability of the sleep duration-BMI relationship across different age groups and study methodologies.

The study’s results reveal a significant correlation between the app-measured midpoint of sleep and both BF% and VAT, but not BMI. This correlation aligns with previous research that recognizes the role of evening chronotype and circadian disruption in excessive body fat accumulation. Our data suggest that this negative effect may be observable in the early stages of obesity development, when weight status (measured by BMI) remains unchanged, but body composition (measured by BF% and VAT) is already deteriorating. The growing body of evidence suggests that the relationship between circadian disruption and obesity is driven by alterations in the hormonal rhythmicity of melatonin, leptin, and glucocorticoids, leading to disruptions in energy homeostasis [47]. Late mealtimes, which often result from circadian misalignment [48], have also been linked to weight gain and obesity [49], with potential underlying mechanisms including decreased energy expenditure during rest and after eating and increased insulin resistance [50]. The fasting-feeding cycle plays a role in synchronizing peripheral circadian rhythms with the central clock [51], while shifting mealtimes can result in or worsen circadian disruption by uncoupling peripheral and central clocks. Recent studies suggest that circadian misalignment can cause imbalances in the gut microbiota, which plays a crucial role in energy homeostasis [52]. This misalignment, represented by lower IS in this study, can result in changes to diurnal fluctuations in the gut microbiota and lead to glucose intolerance and obesity [53]. Additionally, misaligned circadian rhythm has been linked to later mealtimes, higher consumption of calories and saturated fat [54], reduced intake of Mediterranean diet components, and ultimately, a higher BMI [55]. Compared to the actigraphy-measured midpoint of sleep, the app-measured midpoint was found to be 12.7 (SD 43.2) minutes later without statistical significance (*P*=.17). Despite this relatively small difference, it resulted in variations in the relationship between VAT, BF%, and the midpoint of sleep measured by app and actigraphy. This study suggested that the app-measured midpoint of sleep is more clinically sensitive in reflecting obesity indicators, as it is based on human-smartphone interactions and associated smartphone use, which affects melatonin secretion through exposure to smartphone light. Our results suggested that an app-measured chronotype, including sleep and wake times and midpoint of sleep, may provide a more representative measurement of melatonin secretion trends compared to actigraphy, which only captures physical activity. The method used in this study, which incorporates both physical activity and smartphone use, provides a measurement that is closer to melatonin secretion, considered the gold standard in measuring circadian rhythm.

This study has several limitations that should be considered when interpreting the results. First, the cross-sectional design of the study precludes the ability to make causal inferences about the relationship between rest-activity rhythm and health outcomes. Longitudinal studies are needed to further explore these associations and identify the potential mechanisms involved. Second, the methods used to measure sleep duration and circadian rhythm in this study, which are based on human-smartphone interactions, have not been validated against the gold standard measures of polysomnography and melatonin secretion, respectively. However, previous research has found that rest-activity rhythms measured by actigraphy, and mobile apps are associated with the timing of light exposure, the strongest zeitgeber of the circadian system, and the amplitude of melatonin secretion, a classic circadian phase marker [19,56]. This suggests that altered rest-activity rhythms, to some extent, reflect circadian disruption and can serve as a relevant biological marker for circadian function. Third, it should also be noted that this study did not record participants’ timing and energy intake from food, as well as other well-known risk factors for obesity. Fourth, a diagnosis of obstructive sleep apnea (OSA) was found in 5 of the participants in the obesity group. Despite controlling OSA as a confounding factor, our results remained consistent. However, it is critical to acknowledge that OSA is a prevalent comorbidity of obesity and is frequently accompanied by sleep disruption and circadian rhythm misalignment [57]. Further research using larger study populations is necessary to further clarify the role and underlying mechanisms of OSA in the relationship between obesity and circadian rhythm. Fourth, while this study demonstrated that sleep onset and wake times calculated using the Rhythm app did not significantly differ from those recorded by actigraphy, there remained a potential for misjudgment of sleep onset. This scenario could have occurred if an individual fell asleep while using their smartphone, for example, while watching Netflix. In our algorithm, we introduced the “app-count” method, where “app-count” was defined as the sum of minute-by-minute use counts within nonoverlapping 5-minute epochs. Sleep onset was determined as a period of 8 consecutive epochs with a zero app-count, whereas wake time was identified as a period of 6 consecutive epochs with a nonzero app-count. Indeed, this study found that the WASO recorded by the app was longer than that recorded by actigraphy. In future research, it may be necessary to adjust the threshold of zero app-count to achieve more accurate recordings. Finally, we faced potential interruptions in data collection from both the wrist-worn actigraphy device and the smartphone app, primarily due to issues such as low battery power or temporary nonuse by participants. To enhance the robustness and continuity of our data in future research, we plan to synergistically integrate the app with actigraphy. This integration strategy aims to mitigate the challenges associated with individual data collection methods. By using dual recording modes, we anticipate a reduction in the likelihood of concurrent data interruptions. The synergy between these 2 distinct data sources is expected to provide a more comprehensive and nuanced understanding of patient behaviors and circadian rhythms, offering a more holistic perspective in our future studies.

In conclusion, our results reveal that disruptions in the circadian rhythms are linked to higher levels of BMI, VAT, and BF%. These associations appear to hold even when taking into account factors such as sleep duration and levels of daytime motor activity. The strongest associations with obesity were seen in metrics of IS and circadian rhythm regularity, as measured by either a mobile app or actigraphy. These findings suggest that regularity in the circadian rhythm may be a promising target for obesity prevention and body weight management interventions. Future studies should further explore the mechanisms underlying these relationships and develop strategies to improve the regularity of circadian rhythms.

## Abbreviations

BF%: body fat percentage
IS: interdaily stability
IV: intradaily variability
OSA: obstructive sleep apnea
PSQI: Pittsburgh Sleep Quality Index
TST: total sleep time
VAT: visceral adipose tissue area
WASO: wake after sleep onset

## Data Availability

The data sets generated or analyzed during this study are not publicly available due to restrictions imposed by informed consent from participants in this study, which has been approved by the institutional review boards of the Chang Gung Memorial Hospital, but they are available from the corresponding author on reasonable request.
